# Elucidating the functional role of *Mycobacterium smegmatis recX* in stress response

**DOI:** 10.1038/s41598-019-47312-3

**Published:** 2019-07-29

**Authors:** Deepika Prasad, Divya Arora, Vinay Kumar Nandicoori, K. Muniyappa

**Affiliations:** 1 Department of Biochemistry, Indian Institute of Science, Bengaluru, 560012 India; 2 National Institute of Immunology, Aruna Asaf Ali Marg, New Delhi, 110067 India

**Keywords:** Bacterial genetics, Microbiology

## Abstract

The RecX protein has attracted considerable interest because the *recX* mutants exhibit multiple phenotypes associated with RecA functions. To further our understanding of the functional relationship between *recA* and *recX*, the effect of different stress treatments on their expression profiles, cell yield and viability were investigated. A significant correlation was found between the expression of *Mycobacterium smegmatis recA* and *recX* genes at different stages of growth, and in response to different stress treatments albeit *recX* exhibiting lower transcript and protein abundance at the mid-log and stationary phases of the bacterial growth cycle. To ascertain their roles *in vivo*, a targeted deletion of the *recX* and *recArecX* was performed in *M*. *smegmatis*. The growth kinetics of these mutant strains and their sensitivity patterns to different stress treatments were assessed relative to the wild-type strain. The deletion of *recA* affected normal cell growth and survival, while *recX* deletion showed no significant effect. Interestingly, deletion of both *recX* and *recA* genes results in a phenotype that is intermediate between the phenotypes of the *ΔrecA* mutant and the wild-type strain. Collectively, these results reveal a previously unrecognized role for *M*. *smegmatis recX* and support the notion that it may regulate a subset of the yet unknown genes involved in normal cell growth and DNA-damage repair.

## Introduction

To maintain genomic integrity, bacteria have developed a complex network of DNA-damage response (DDR) pathways to sense, respond to, and repair different types of DNA damage^1–3^. In *Escherichia coli*, DNA damaging agents induce the SOS regulon - an adaptive stress response - that contributes to cell survival. Most notably, SOS induction increases the mRNA and protein levels of approximately fifty genes belonging to the SOS regulon through RecA-dependent autocatalytic cleavage of the LexA repressor^1,4,5^. Multiple investigations have established that mutations in *E*. *coli recA* are pleiotropic, affecting not only homologous recombination (HR) but also DNA repair, DNA replication, mutagenesis and cell division through the SOS response^1,4,6,7^. These crucial functions are carried out by a helical RecA nucleoprotein filament through three distinct, but related biological processes: (a) recombination-based DNA repair; (b) transcriptional upregulation of SOS genes through cleavage of the LexA repressor and (c) error-prone replication by DNA polymerase IV^4,5^.

In *E*. *coli*, a variety of accessory proteins regulate RecA function and amongst these the single-stranded binding (SSB) protein and RecFOR abet RecA in the formation of nucleoprotein filaments on single stranded DNA (ssDNA)^4,7,8^. Two different types of accessory factors, referred to as RecA mediators and RecA modulators, act at different levels to regulate the formation and/or stability of the RecA-ssDNA filament^8–10^. The RecA mediators act during the binding of RecA on SSB-coated ssDNA. In contrast, the RecA modulators regulate the function of the nucleoprotein filament during the search for homology and/or strand exchange. The well characterized RecA mediators, SSB, RecF, RecO and RecR, act in overcoming the barrier to RecA filament nucleation and stimulate the rate of its polymerization^4,7,8,11^. Differing from these mediators, DinI and RecX proteins modulate RecA protein function but have very different effects^12,13^. Whilst DinI stabilizes RecA nucleoprotein filaments, RecX binds to RecA in solution and also inhibits RecA nucleoprotein elongation and its biochemical functions^14,15^. Other known negative modulators include RdgC and PsiB: whereas RdgC acts as a negative regulator of RecA protein functions, PsiB binds to the RecA that is free in solution and the resultant complex impedes the formation of RecA nucleoprotein filaments^16–19^. Another accessory factor, RecN, a bacterial SMC (Structural Maintenance of Chromosome)-like accessory protein, stimulates the DNA strand-invasion step of RecA-mediated recombination-based DNA repair^20^. However, the functional and mechanistic properties of RecA accessory factors in mycobacteria are not well understood.

The RecX protein has attracted much interest because the *recX* mutants exhibit multiple phenotypes associated with RecA functions. The *Mycobacterium tuberculosis* RecX was first identified for its ability to suppress DNA pairing and strand exchange, ATPase and co-protease activities of RecA^14^. Unsurprisingly, the *E*. *coli* and *Deinococcus radiodurans* RecX proteins were also shown to modulate the biochemical activities of their cognate RecA proteins in a similar fashion^13,15,21^. However, the mechanisms by which RecX affect the biochemical activities of RecA remain controversial and are still being defined. For instance, *E*. *coli* RecX, which is far less abundant in the cell, interacts with DNA but it is not clear how such an interaction relates to its capacity to inhibit RecA activities. Furthermore, the mechanism by which RecF antagonizes the *E*. *coli* RecX promoted destabilization of RecA nucleoprotein filaments is not well understood^22^. The electron microscopy reconstructions of mixed RecA-RecX filaments revealed that RecX binds within the helical groove of the RecA nucleoprotein filament^23^. As a result, a repression of HR might ensue by the steric clash of RecX with the process of DNA strand exchange and impede the ATP-coupled allosteric changes in RecA^14,23,24^. Studies in *Bacillus subtilis* suggest that RecX facilitates HR by modulating RecA activities by inhibiting RecA^25^ and RecX aids all RecA-related processes in *Neisseria gonorrhoeae*^26^. Other studies have found that mechanical forces antagonize the inhibitory effects of *M*. *tuberculosis* RecX, and a partially de-polymerized RecA filament could re-polymerize in the presence of RecX^27^. Although these findings shed light on the role of RecX, the molecular mechanisms that underlie the variety, magnitude and taxa specific activities are not fully understood.

Moreover, very little is known about the *in vivo* functional relationship between *recA* and *recX*, especially in intracellular bacterial pathogens. *M*. *tuberculosis* is the causative agent of tuberculosis, which is characterized by slow growth. Therefore, *M*. *smegmatis* is often used as a surrogate model to study the pathogenesis of mycobacterial infection and drug resistance. Further, investigations into the interaction mechanisms between *M*. *smegmatis recX* and *recA* might reveal insights into the phylogenetic diversification among the mycobacterial species. To this end, *M*. *smegmatis ΔrecX* and *ΔrecAΔrecX* mutants were constructed and subjected to various stress conditions. The growth phenotypes of these mutants and *recA* and *recX* gene expression profiles were compared with those of the wild-type strain. In the absence of DNA damage, *recX* was found to be non-essential for *M*. *smegmatis* growth, and *ΔrecAΔrecX* double mutant strain exhibits a phenotype that is intermediate between the phenotypes of the *ΔrecA* mutant and the wild-type strain. Additionally, a significant correlation was seen between the expression of the *recA* and *recX* at different stages of growth and after exposure to DNA damaging agents, albeit *recX* exhibiting lower transcript and protein abundance at the mid-log and stationary phases of growth. Collectively, these results suggest a previously unrecognized role for *M*. *smegmatis recX* and support the idea that it may regulate a subset of the yet unknown genes involved in normal cell growth and DNA damage repair.

## Methods

### Chemicals and enzymes

All the chemicals were purchased from Sigma-Aldrich (St. Louis, MO). All restriction endonucleases, Phusion polymerase, Klenow polymerase and T4 DNA ligase were purchased from New England Biolabs (Ipswich, MA). Pure H_2_O_2_, methylmethane sulphonate (MMS), ciprofloxacin, kanamycin and hygromycin were purchased from Sigma-Aldrich (St. Louis, MO). RT-qPCR reagents, SYBR RT-qPCR master mix and cDNA synthesis kit were obtained from Bio-Rad Laboratories (Hercules, CA). The DNA oligonucleotides were synthesized by Sigma-Genosys (Bangalore, India) and [α-^32^P]ATP was purchased from the Board of Radiation and Isotope Technology (Hyderabad, India).

### Bacterial strains, plasmids and growth conditions

Bacterial strains and plasmids used in this study are described in Table 1. The *M*. *smegmatis* mc^2^155 *ΔrecX* and *ΔrecAΔrecX* strains were generated as described^28^. The pJV53 vector (encoding mycophage derived recombinases under acetamide-inducible promoter was transformed into *M*. *smegmatis* mc^2^155^28^. The *ΔrecAΔrecX* and *ΔrecX* mutants were derived from *M*. *smegmatis* mc^2^155 strain. These strains were grown in liquid 7H9 Middlebrook broth (Difco Laboratories, Detroit, MI) supplemented with 0.05% Tween 80 and 10% albumin-dextrose-catalase (ADC) (henceforth referred to as 7H9 broth) with continuous agitation (120 rpm) at 37 °C for 2 days. *M*. *smegmatis* mc^2^155 was grown on 7H10 agar (4.7 g/L) plates supplemented with 10% ADC. The starter cultures were prepared by inoculating a single colony of *M*. *smegmatis* mc^2^155 into 15 ml of 7H9 broth and grown at 37 °C in a shaking incubator (120 rpm), until the cultures reached an OD_600_ of 1. Then 1.5 ml of the cell suspension was inoculated into 30 ml of 7H9 Middlebrook medium to give an OD_600_ of 0.05. The antibiotics (kanamycin and hygromycin) were added to a final concentration of 25 µg/ml and 100 µg/ml respectively.

**Table 1:** Bacterial strains and plasmids used in this study.

Plasmids or strains	Relevant characteristics (genotype and phenotype)	Reference or source
pJV53	Plasmid encoding phage Che9c 60 and 61 genes under acetamide-inducible promoter; *kan*^*r*^	(^28^)
pVV16	Shuttle vector (*E*. *coli* and *M*. *smegmatis*) used for expression of genes in *M*. *smegmatis*, *kan*^*r*^	(^29^)
p0004S	Vector backbone used for the cloning of allelic exchange substrate of *ΔrecA ΔrecX* and *ΔrecX* strains	(^29^)
*ΔrecA*	*M*. *smegmatis* mc^2^155 disrupted for *recA* gene, *Hyg*^r^, retarded growth under normal growth condition, severe growth defect under DNA damaging conditions	(^50^)
*ΔrecA ΔrecX*	*M*. *smegmatis* mc^2^155 disrupted for *recA* and *recX* genes, *Hyg*^*r*^, growth phenotype intermediate of WT and *ΔrecA* strains	This study
*ΔrecX*	*M*. *smegmatis* mc^2^155 disrupted for *recX* gene, *Hyg*^*r*^, no phenotype	This study
*M*. *smegmatis* mc^2^155 recombineering strain	*M*. *smegmatis* mc^2^155 electroporated with pJV53 vector	This study

The *M*. *smegmatis ΔrecA* mutant was a kind gift from Elaine O. Davis, National Institute for Medical Research, London. The characteristics of plasmids (pJV53, pVV16 and p0004S) have been described elsewhere^29^. The bacterial cultures were exposed to a UV lamp (UVP Inc. San Gabriel, CA) at 254 nm for different time intervals ranging from 5 to 20 sec. The dosage was measured using a radiometer (UVItec Ltd, Cambridge, UK).The *E*. *coli* DH5α strain (Novagen, Madison, WI) was used in the recombinant DNA experiments. The cultures were incubated at 37 °C with shaking at 120 rpm, and OD_600_ values were recorded every 3 h, until the cultures reached an OD_600_ of 2.5.

### Cloning of *recA* and *recX* genes

*M*. *smegmatis recA* (MSMEG_2723) *and recX* (MSMEG_2724) ORFs were obtained from the SmegmaList website (http://svitsrv8.epfl.ch/mycobrowser/smegmalist.html). The coding sequences of *recA* and *recX* were amplified by PCR using gene-specific forward and reverse primers (Table 2). The pVV16-vector was digested with NdeI and HindIII. The PCR products were gel purified, and ligated separately into the linear plasmid pVV16 DNA at

**Table 2:** The sequences of primers for PCR-amplification of *M*. *smegmatis recA* and *recX* ORFs.

Primers	Oligonucleotide Sequence (5′ → 3′)	Restriction Enzyme
Ms*recA* (FP)	AGCATACATATGGCGCAGCAGGCC	NdeI
Ms*recA* (RP)	GATTATAAGCTTTCAGAAGTCAAC	HindIII
Ms*recX* (FP)	TCCTCATATGACGTCCTCCCGGCCCCG	NdeI
Ms*recX* (RP)	AATTACAAGCTTCTAGACCCGCCGGC	HindIII

(RP and FP stand for reverse and forward primers respectively).

NdeI/HindIII site. The resulting plasmids contained either full-length *M*. *smegmatis recA or recX* genes under the control of the Hsp60 constitutive promoter.

### Complementation assay

The complementation assay was performed using *M*. *smegmatis* mc^2^155 *ΔrecA*, *ΔrecAΔrecX* and *ΔrecX* mutant strains. The plasmid complemented strains bearing wild-type copies of *M*. *smegmatis recA* or *recX* ORFs were created by electroporation. These were grown in liquid 7H9 broth to mid-log phase, until cultures reached an OD_600_ of 0.6. The cultures were centrifuged and pellets were resuspended to an OD_600_ of 0.8 in sterile Milli-Q water. An aliquot from each culture was serially diluted and spotted on 7H10 agar plate containing 100 µg/ml of hygromycin B. The cells were exposed to 10 J/m^2^ of 254 nm UV light and incubated at 37 °C in the dark for 3 days. The images were acquired in the digitization mode using a Quant LAS 4000 imaging system (GE Healthcare, Piscataway, NJ).

### Construction of *recX* and *recA-recX* gene replacement mutants in *M*. *smegmatis*

The DNA manipulations were carried out using standard protocols^30^. Plasmid DNA and DNA fragments were isolated from the gels using Qiagen miniprep kit (Qiagen India, New Delhi) according to the manufacturer’s instructions. The genomic DNA of *M*. *smegmatis* was PCR-amplified as described^31^. The electrocompetent *M*. *smegmatis* mc^2^155 cells were prepared and stored as described^32^. The construction of allelic exchange substrate (AES) and its subsequent delivery to the recombineering strain of *M*. *smegmatis* mc^2^155 was carried out as described^28^. Table 3 shows the sequences of upstream and downstream primers corresponding to the *M*. *smegmatis recA-recX* locus. For *recX* gene deletion, the upstream and downstream flanking regions of *M*. *smegmatis* mc^2^155 *recX* were PCR-amplified from the genomic DNA using specific primers that contained the restriction sites in the counter-selectable vector p0004S. The upstream and downstream flank fragments were digested with PflMI and directionally cloned on either side of the Hyg resistance gene to generate the *recX*-AES. To generate allelic exchange substrates, the upstream and downstream flanking sequences of *recA-recX* locus were PCR-amplified using genomic DNA as the template and specific primers. The amplified products were digested with PflMI and directionally cloned on either side of the Hyg resistance gene. The resulting constructs were independently transformed into *E*. *coli* DH5α and plated on LB agar containing 100 μg/ml of hygromycin. The positive clones were identified by restriction analysis of the isolated plasmid using EcoRV to generate the linear blunt-ended recombineering-proficient (containing upstream region-*hyg*^*r*^-downstream region) DNA fragments.

**Table 3:** The sequences of oligonucleotides used for the construction of AES.

Sl. no.	Primers	Oligonucleotide sequence (5′ → 3′)
1	*recA* 5′ flank FP	CACCTTTT**CCATAAATTGG**GATATCGCCGGCCGCGCGGATCCGGTCG
2	*recA* 5′ flank RP	TTTTTTTT**CCATTTCTTGG**GGCTGGCGCACCTCTTCGCCG
3	*recX* 5′ flank FP	CACCTTTT**CCATAAATTGG**GATATCCTCGAACTGGCGATGGCCCAG
4	*recX* 5′ flank RP	TTTTTTTT**CCATTTCTTGG**TCAGAAGTCAACCGGGGCCGG
5	*recX* 3′ flank FP	TTTTTTTT**CCATCTTTTGG**GATAT CGCCGGGGCCACGCGCGCGGG
6	*recX* 3′ flank RP	TTTTTTTT**CCATCTTTTGG**GATATCGCCGGGGCCACGCGCGCGGG

(RP and FP stand for reverse and forward primers respectively).

Note: the sequences shown in bold are the recognition sites of PflMI and those underlined are for EcoRV. Primer pairs 1, 2 and 3, 4 were used for the amplification of upstream region of*recA* and *recX* genes, respectively. Primers 5 and 6 were used to amplify downstream region of *recX* gene.

### Preparation of *M*. *smegmatis* mc^2^155 recombineering strain and construction of *ΔrecX* and *ΔrecAΔrecX* strains

The *M*. *smegmatis* mc^2^155 derivative harbouring the pJV53 vector was used in recombineering^28^. The AES constructs generated for disruption of *recX* and *recArecX* were linearised using EcoRV and then isolated by electroelution. The linearized AES constructs (100 ng each), were independently electroporated into the *M*. *smegmatis* mc^2^155 recombineering strain. The transformed cells were cultured with shaking at 37 °C for 4 h. The transformants were plated on 7H10 agar plates, supplemented with 100 μg/ml hygromycin B and 25 µg/ml kanamycin. The transformants were selected and their genomic DNA was isolated and analyzed by PCR to ascertain recombination in the *M*. *smegmatis* mc^2^155 genome at the allelic site.

### Southern blot hybridization

The genomic DNA from the *M*. *smegmatis* mc^2^155 wild-type, *ΔrecA* and *ΔrecAΔrecX* strains was isolated to confirm deletion of the *recX* and *recArecX* genes^31^. The probe (548 bp) corresponding to the upstream region of *recA* was PCR-amplified using forward (5′ GCTCACCTCCATCGACAGGATCCT 3′) and reverse primers (5′ GGTGCCTCTCCGAGTAGTCGTGTC 3′). The PCR amplified product (labelled probe) was prepared using [α-^32^P]ATP, random primers (hexamer) and Klenow polymerase as described (30). The radiolabeled probe was heat denatured at 95 °C for 10 min followed by snap-chill prior to use. Approximately 10 µg of genomic DNA was digested using NdeI and SalI (10 U each) at 37 °C overnight and the DNA fragments were separated by 0.8% agarose gel electrophoresis. The gel was soaked in 0.25 N HCl for 20 min followed by washing twice with 40 ml of sterile water. Subsequently, the gel was submerged in a solution containing 0.5 N NaOH and 0.6 M NaCl for 15 min each to denature the DNA, followed by neutralization of the gel pH in a buffer containing 1.5 M NaCl and 0.5 M Tris-HCl (pH 7.5) for 30 min, and finally in 10X SSC (pH 7). The DNA was transferred by capillary action to a nylon membrane and was fixed to dry membranes by exposure to UV light at 254 nm using a Stratalinker (Stratagene Corporation, La Jolla, CA). The nylon membrane was soaked in a pre-hybridization buffer (0.5 M phosphate buffer pH 7, 7% SDS, 1 mM EDTA) by gentle shaking for 1 h at 65 °C and then hybridized using the ^32^P-labelled probe at 65 °C for 16 h. Following hybridization, the membrane was washed with 1X SSC and 0.1% SDS to remove the unbound probe. The bands were visualised using the Fuji FLA 9000 phosphorimager.

### Analysis of *M*. *smegmatis recA* and *recX* gene expression under hypoxic conditions

Wayne and Hayes dormancy culture system was used to study the hypoxic response of *M*. *smegmatis recA* and *recX* expression as described^33,34^. Briefly, *M*. *smegmatis* mc^2^155 strain was grown as described above until the culture reached an OD_600_ of 0.6. An aliquot of the culture was diluted in Middlebrook 7H9 broth to an OD_600_ of 0.05. Equal volumes (5 ml) of diluted culture were placed in each of 100 ml screw-cap flasks having a headspace ratio of ~ 0.5 (35 ml of head space air vol. to 65 ml of liquid medium) containing a stir bar. The cultures were gently shaken (120 rpm) at 37 °C. To ascertain hypoxic culture conditions, we used a standard color assay (viz., methylene blue reduction)^35^. Methylene blue (1.5 μg/ml) was added to cultures, and reduction was monitored visually (by decolorization). To avoid aeration of cultures during sampling, separate flasks were used for each time point in these experiments. A small portion of the culture from each flask was used for Western blot analysis, while the rest was used for total RNA extraction and RT-qPCR as described below.

### DNA damage-sensitivity assays

The *M*. *smegmatis* mc^2^155 wild-type, *ΔrecA*, *ΔrecX* and *ΔrecAΔrecX* strains were grown in 7H9 broth to mid-log phase to a cell density of 0.6. After 3 h of incubation with different concentrations of ciprofloxacin, MMS or hydrogen peroxide, the cells were collected by centrifugation and resuspended in 1 ml sterile Milli-Q water at a cell density of 0.8. A portion of the resuspended cells were serially diluted and spotted on 7H10 agar plates containing 100 µg/ml hygromycin B. Similarly, the serial dilutions of wild-type and mutant strains were plated on 7H10 agar and irradiated under 254 nm UV light. The plates were incubated at 37 °C in the dark. The images were acquired in digitization mode using the Quant LAS-4000 chemidocumentation system (GE Healthcare Life Sciences, Pittsburgh). In parallel experiments, serial dilutions of cell cultures, treated with the indicated concentrations of MMS, H_2_O_2_, or ciprofloxacin, were spread on 7H10 agar plates containing 100 µg/ml hygromycin. The survival was determined by counting viable cells.

### RNA isolation and quantitative real-time PCR

The expression of *recA* and *recX* in *M*. *smegmatis* mc^2^155 cells, grown under aerobic or hypoxic conditions, were determined by quantitative real-time PCR using specific primers (Table 4). The cultures were harvested at the time points corresponding to early-log, exponential and stationary phases. The cell pellets were suspended in 5 ml of freshly prepared protoplast buffer (15 mM Tris-HCl, pH 8, 0.45 M sucrose, 8 mM EDTA and 4 mg/ml lysozyme) and incubated at 37 °C for 1 h. The suspension was centrifuged at 4000 rpm for 10 min at 4 °C, and the resulting pellet was resuspended in 1050 µl of RLT buffer (Qiagen India, New Delhi). The total RNA was isolated from the whole cell lysates using the Qiagen RNeasy-kit as per the manufacturer’s instructions. The RNA preparations (1.4 µg in 20 µl) were incubated with DNase I (1 U/µg) at 37 °C for 1 h. The RNA samples (0.7 µg) were denatured at 65 °C for 10 min and were used in cDNA synthesis utilizing the iScript cDNA synthesis kit (Bio-Rad Laboratories, Hercules, CA) according to the manufacturer’s instructions. The RNA samples without reverse transcriptase were included as a negative control.

**Table 4:** The sequences of primers used for RT-qPCR.

Primers	Oligonucleotide sequence (5′ → 3′)
*recA* FP	CTGAAGTTCTACGCCTCGGT
*recA* RP	AGACCTTGTTCTTGACGACCT
*recX* FP	GTTGGTGCGCGACAAGTT
*recX* RP	CATCGACTGGTTGTATCCGC
*sigH2* FP	CTCAAGGCGTACAACGGTTT
*sigH2* RP	TCCTCACACAACTGCTCGAC
*sig*H7 FP	CGTGTACTACGCCTGTGTCG
*sig*H7 RP	CTGGTCGTTATCGGTCGAAT
16S rRNA FP	CTGAGATACGGCCCAGACTC
16S rRNA RP	CGTCGATGGTGAAAGAGGTT
MSMEG_2725 FP	ATCGGCTACCAGATCACGTT
MSMEG_2725 RP	CGCAAAGTTCACGCTGATCA
MSMEG_2726 FP	CAACCATCCAACTGACGGTG
MSMEG_2726 RP	TCCCCATCCAGTTCATCACC
MSMEG_2720 FP	TTTCCTCGCCTGGTTCTTCA
MSMEG_2720 RP	CGCACCAATACCCTTCGTTC

(RP and FP stand for reverse and forward primers respectively).

The cDNA synthesis was performed using the following parameters: 25 °C for 10 min, 42 °C for 30 min followed by termination at 85 °C for 5 min. The RT-qPCRs (in 20 µl) were carried out with 2 ng of cDNA template, 10 µl of SYBR green (2X), 0.5 µl of both forward and reverse primers (0.25 µM each) in the BioRad iQ5 multicolor real time PCR detection system. The cycling program comprised initial denaturation at 95 °C for 10 min, denaturation at 95 °C for 15 sec, primer annealing at 60 °C for 30 sec and extension at 72 °C for 30 sec. Following amplification, a melting curve analysis was performed to verify the authenticity of the amplified product. Similarly, the levels of MSMEG_2725, MSMEG_2726 and MSMEG_2720 ORFs in *M*. *smegmatis* mc^2^155 wild-type, *ΔrecA ΔrecX* and *ΔrecX* mutant cells were determined by RT-qPCR.

### Western blot analysis

The whole cell lysates of *M*. *smegmatis* mc^2^155 cells, grown under aerobic or hypoxic conditions, were prepared as described above (see under RNA isolation and RT-qPCR). The antibody against GroEL was purchased from Sigma-Aldrich (St. Louis, MO). The polyclonal antibodies against *M*. *tuberculosis* RecA and RecX were raised in rabbits and characterized as described^14^. The whole cell lysate (30 µg of protein) was boiled in SDS/PAGE buffer for 10 min, and the proteins were separated by SDS-PAGE using 10% (wt/vol) polyacrylamide gels as described^36^. After gel electrophoresis, the proteins were transferred onto a polyvinylidene difluoride membrane. The membranes were incubated with either anti-RecA, anti-RecX or anti-GroEL antibodies at a dilution of 1:15000, 1:1000 and 1:80000 respectively as described^37^. The membranes were blocked with 3% BSA in phosphate buffered saline (137 mM NaCl, 2.7 mM KCl, 10 mM Na_2_HPO_4_ and 1.8 mM KH_2_PO_4_) and washed with TBST (50 mM Tris-HCl, pH 8, 150 mM NaCl and 0.1% Tween 20). Subsequently, the membranes were incubated with peroxidase-conjugated (Sigma-Aldrich, St. Louis, MO) secondary antibody. The blots were developed using luminol and hydrogen peroxide. The digital images of Western blot bands were acquired using the Quant LAS-4000 chemidocumentation system (GE Healthcare Life Sciences, Pittsburgh, PA), and quantified using the ImageJ software.

### Statistics and data presentation

Statistical significance was determined using the unpaired t-test. P-value (p) less than 0.05 indicate significant difference and p > 0.05 is not significant (ns). The symbols *, ** and *** correspond to p < 0.05, p < 0.01 and p < 0.001 respectively.

## Results and Discussion

### Hypoxia causes an increase in the expression of *M*. *smegmatis recA* and *recX* genes

During the course of infection and proliferation, *M*. *tuberculosis* is exposed to physiological stress conditions such as hypoxia and acidic pH stress, which could compromise its survival^38–40^. For instance, acute hypoxic stress arrests DNA replication and triggers robust DNA damage^41–43^. An increase in the expression of genes of the SOS pathway, including *umuDC*, *polB*, *recN*, *sulA*, *uvrA*, *uvrB*, and *uvrD*, begin upon DNA damage^44–46^. To this end, hypoxia-induced gene expression profiling in *M*. *smegmatis* and *M*. *tuberculosis* during latent infection has provided valuable insights on the nature of latent tubercle bacilli and its treatment^47^.

Previous studies in *M*. *tuberculosis*^48^, *Thiobacillus ferrooxidans*^49^ and *Streptomyces lividans*^50^ have demonstrated that *recA* is co-transcribed with *recX*. Furthermore, an overexpression of RecA (a positive regulator of SOS response) was found to be toxic in the absence of RecX in certain bacterial species including *Pseudomonas aeruginosa*^51^, *S*. *lividans*^50^, *M*. *smegmatis*^52^, and *Xanthomonas oryzae*^53^. These findings are consistent with the notion that RecX acts as an anti-recombinase to quell inappropriate recombination events promoted by RecA^14^. In contrast, an overexpression of RecA in the *E*. *coli ΔrecX* strain is not deleterious and the deletion of *recX* does not affect *recA* expression^54^. The *recX* transcription in *E*. *coli* is downregulated compared to *recA* under normal growth conditions because of the existence of a transcription termination site between the *recA* and *recX* coding sequences. However, a *recA-recX* mRNA transcript resulting from transcriptional read-through accumulates in the range of 5–10% of the total *recA* mRNA content^54^. The *recX* transcript was hardly detectable during the vegetative growth of *E*. *coli*, but robust expression of *recX* occurs following treatment with DNA damaging agents^15,55^.

**Figure 1:**
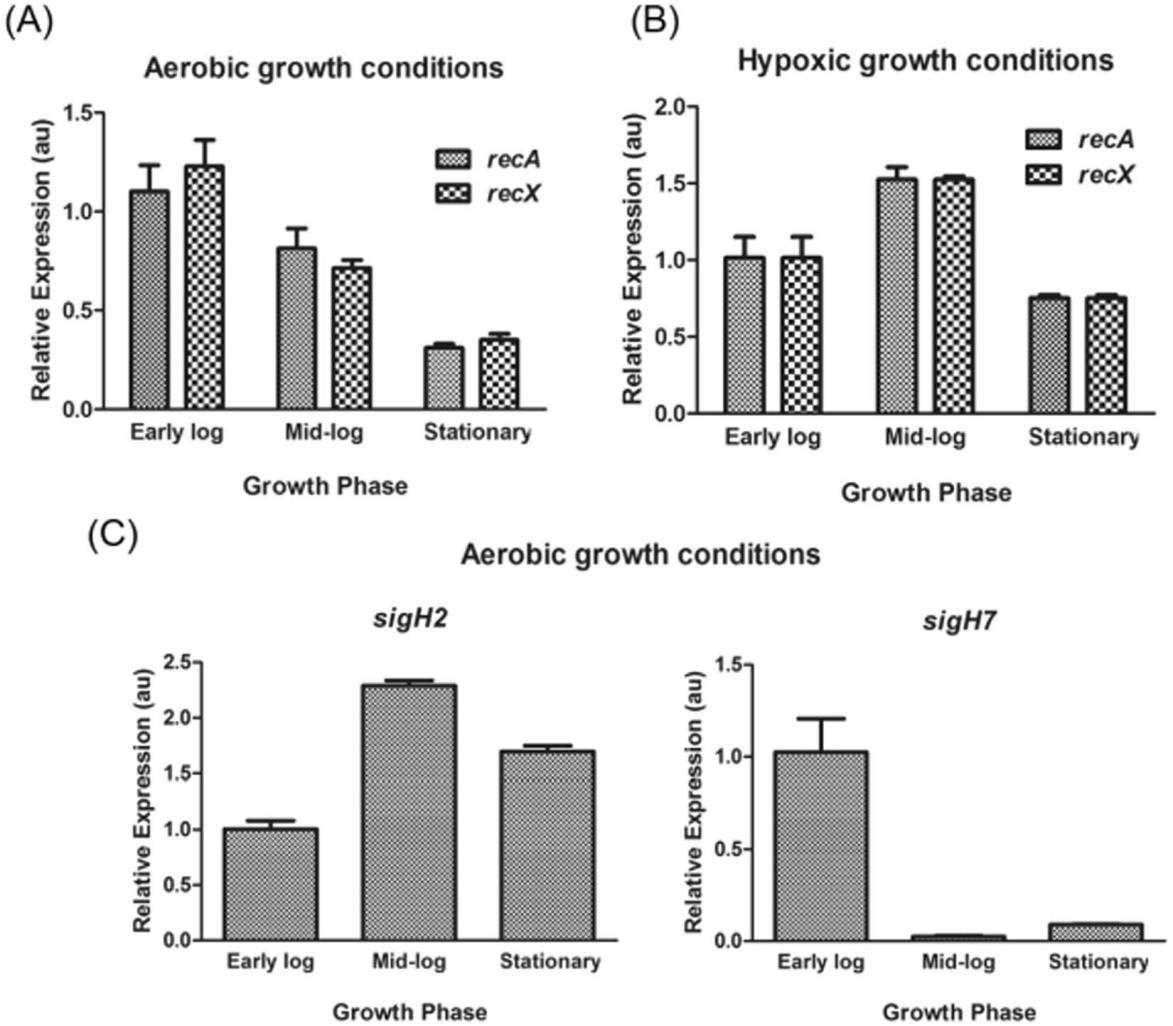
(**A**,**B**) Relative levels of expression of *M*. *smegmatis* mc^2^155 *recA* and *recX* genes at the early log, mid-log and stationary phases under aerobic and hypoxic growth conditions. (**C**) Relative levels of expression of *sigH2* and *sigH7* genes at the early log, mid-log and stationary phases. The signal intensities were determined as described in the Methods section. The histograms represent the mean values of three independent experiments. The expression levels were determined and normalised to 16S ribosomal RNA expression and induction ratios calculated relative to the amount of transcript in the aerobic early log phase culture. The error bars represent the standard error of the mean calculated from 3 independent replicates.

The genomes of both *M*. *smegmatis* and *M*. *tuberculosis* contain a single copy each of *recA* and *recX* in a single operon. To investigate the expression and regulation of these genes under aerobic and hypoxic growth in *M*. *smegmatis*, a commonly used surrogate model for *M*. *tuberculosis*, the relative abundance of mRNA at various growth phases was measured by a RT-qPCR assay and normalized to that of the constitutively expressed 16S rRNA gene. The cycle threshold (Ct) values for *M*. *smegmatis recA* and *recX* mRNA were normalized against the Ct value of the16S rRNA gene. In contrast to *E*. *coli*^15,55^, significant amount of *recA* mRNA transcripts were seen at early-log phase under both aerobic and hypoxic culture conditions (Fig. 1A). Interestingly, hypoxic conditions enhanced the accumulation of *recA* mRNA by 1.5-fold at mid-log phase, and decreased thereafter as the cells entered the stationary phase. A similar pattern was observed with *recX* mRNA abundance under both aerobic and hypoxic conditions, albeit at reduced levels (Fig. 1B). These observations support the idea that the two genes are co-transcribed and coordinately regulated under aerobic and hypoxic growth conditions. Intriguingly, no difference was seen in the amounts of mRNA under aerobic and hypoxic growth conditions at the early-log phase. The transcriptional profiles of *M*. *smegmatis* growth-phase-specific marker genes (*sigH2* and *sigH7*)^56^ was used to ascertain the times of the early-log, the mid-log and the stationary phases (Fig. 1C). In multi-drug resistant clinical strains of *M*. *tuberculosis*, the levels of both *recA* and *recX* mRNA were found to be higher than in the drug-susceptible strains and the levels of *recX* mRNA increased concomitantly with a rise in *recA* mRNA^57^.

**Figure 2:**
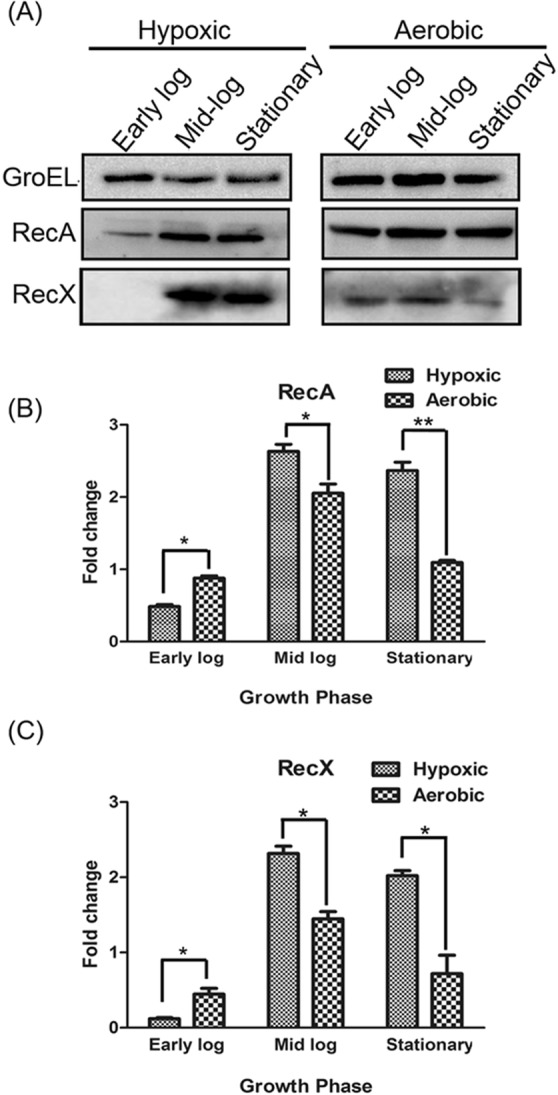
(**A**) Expression levels of *M*. *smegmatis* mc^2^155 RecA and RecX proteins under aerobic and hypoxic growth conditions. (**B**) The quantification of relative changes in RecA protein levels under aerobic and hypoxic growth conditions. (**C**) The quantification of relative changes in RecX levels under aerobic and hypoxic growth conditions. The histograms represent the mean values determined by quantification of Western blots from three independent experiments. The loading control GroEL was used for normalization of RecA and RecX expression levels. The error bars represent the standard error of the mean calculated from 3 independent replicates. Significant differences are indicated by **p < 0.01, and *p < 0.05. ns, not significant.

However, it should be noted that the mRNA levels cannot be used as surrogates for the corresponding protein levels. Therefore, the abundance of RecA and RecX proteins in *M*. *smegmatis* was measured under aerobic and hypoxic conditions by a Western blot assay using anti-RecA and anti-RecX antibodies. GroEL was probed as a protein-loading control. The densitometric analysis of immunoblots revealed that cells accumulated higher levels of RecA under hypoxic growth conditions compared to aerobic conditions (Fig. 2A,B). A comparative analysis showed that the cells consistently accumulated 2-fold higher levels of RecA at mid-log phases relative to the early-log phase under aerobic growth conditions, which decreased at the stationary phase to levels seen at the early-log phase (Fig. 2A,B). A similar pattern was observed for RecX, albeit the levels were less pronounced than that of RecA (Fig. 2A,C). Although the mRNA and protein expression patterns were comparable, important differences were noted. First, the RecX protein was hardly detectable at early-log phase during hypoxic conditions. Second, the abundance of RecA mRNA and protein were higher compared to RecX.

Although, these results suggest that *M*. *smegmatis recA* and *recX* genes are induced under hypoxic conditions, there is no correlation between the amount of *recX* mRNA and corresponding protein in the early-log phase. Both *M*. *tuberculosis* and *M*. *smegmatis* have been shown to stabilize their mRNA transcripts under growth-inhibiting conditions^58–60^. Among possible mechanisms, tRNA reprogramming and selective codon-biased translation have been shown to play a role in mycobacteria in response to hypoxia^61^. One possible explanation for the lack of correlation between the *recX* mRNA versus protein level in the early log phase could be due translational repression of *recX* mRNA.

### Construction of *ΔrecX* and *ΔrecA ΔrecX* mutant strains

The difference in the RecA and RecX protein levels in *M*. *smegmatis* indicate possible regulation of gene expression at the level of transcription. In many bacteria studied so far, the *recX* gene is located on the same coding strand downstream of *recA* and the two genes are co-transcribed^48,50,53,54,62,63^. However, in the *lexA-recA-recX* locus of *Xanthomonas* pathovars, each gene is expressed from its own promoter^64^. Furthermore, in *D*. *radiodurans*^65,66^, *B*. *subtilis*^67^ and *N*. *gonorrhoeae*^26^, the *recA* and *recX* genes are separated by several hundred kb in length and transcribed from their own promoters.

In a number of mycobacterial species, as well as in a few other bacteria, the 5′ region of the *recX* coding sequence overlaps the 3′ region of the *recA* gene. The overlap is 32 bp long in *M*. *smegmatis*^48^, while in *M*. *tuberculosis*^68^ and *M*. *leprae*^69^ the overlap is 35 bp. The effect of *recX* deletion on the phenotypic characteristics has been studied in various organisms^7,10^. The knockout mutants of *recX* show a range of phenotypes associated with RecA functions: the inactivation of *B*. *subtilis recX* rendered the cells sensitive to MMS and H_2_O_2_^25^, the *S*. *lividans recX* mutants displayed decreased resistance to UV damage^42^ and the *N*. *gonorrhoeae recX* mutant showed a small decrease in its ability to survive DNA damage that was caused by double-strand breaks^26^. However, the impact of deletion of both *recA* and *recX* genes on the growth characteristics and DNA damage repair has not been investigated in any organism. Moreover, a full understanding of the *in vivo* role of *recX* in mycobacteria is not fully understood.

**Figure 3:**
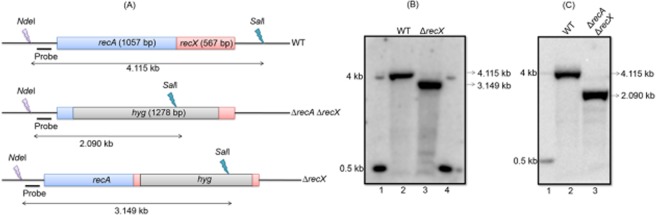
Isolation of *M*. *smegmatis* mc^2^155 *ΔrecX* and *ΔrecA ΔrecX* mutant strains. (**A**) physical map of the *M*. *smegmatis recA recX*, *ΔrecAΔrecX* and *ΔrecX* regions. The horizontal lines with arrowheads on both ends represent the size of the genomic DNA fragment between NdeI and SalI restriction sites corresponding to the wild-type, *ΔrecA ΔrecX* and *ΔrecX* strains. The lightning bolt symbols indicate recognition sites for the indicated restriction enzymes. The little black boxes (adjacent to the NdeI recognition site) indicate hybridization probes used in Southern blotting experiments. (**B**) Southern blot analysis of genomic DNA from the wild-type and *ΔrecX* strains. Lane 1 and 4, molecular weight markers; 2, genomic DNA from the wild-type strain; 3, genomic DNA of the *ΔrecX* strain. (**C**) Southern blot analysis of genomic DNA of the wild-type and *ΔrecAΔrecX* strains. Lane 1, molecular weight markers; 2, genomic DNA of the wild-type strain; 3, genomic DNA of the *ΔrecAΔrecX* double mutant strain.

Previous studies have demonstrated that the *M*. *smegmatis ΔrecA* strain exhibited sensitivity to UV-induced DNA damage and fails to promote HR^52^. We combined the *recA* mutation with the deletion of *recX* to investigate the effects of the double mutations. For this purpose, *M*. *smegmatis* mc^2^155 *recX* single and *recArecX* double mutant strains were generated. As a control, the effect of *recA* deletion in *M*. *smegmatis* was re-evaluated for direct comparison. Using the recombineering method, which is based on a protocol developed for *M*. *tuberculosis* and *M*. *bovis* BCG^28^, the *M*. *smegmatis* mc^2^155 *ΔrecA* and *ΔrecAΔrecX* mutants were generated using 3.279 kb and 3.302 kb linear *ΔrecA::hyg* and *ΔrecAΔrecX::hyg* AES constructs, respectively. Approximately 100 ng of linear AES DNA fragments were generated by restriction digestion of the respective plasmids with EcoRV (Fig. 3A). These were transformed into competent *M*. *smegmatis* mc^2^155:pJV53 recombineering cells^70^. After 4–5 days of incubation, 8–10 Hyg-resistant colonies were found for the *ΔrecX* and *ΔrecAΔrecX* mutants. The putative mutant strains were screened using PCR to determine if the *recX* and *recArecX* genes were deleted from the chromosome (data not shown). The correct mutants were grown in 7H10 Middlebrook agar medium for 5–8 generations to allow the loss of pJV53.

### Genotypic and phenotypic analysis of *M*. *smegmatis ΔrecX* and *ΔrecAΔrecX* mutants

After screening by PCR, 2 each of the *ΔrecX* and *ΔrecAΔrecX* knockout mutants of *M*. *smegmatis* mc^2^155 were obtained. The *ΔrecX* and *ΔrecAΔrecX* mutants were characterized by restriction enzyme mapping and Southern blot hybridization (Fig. 3). Upon hybridization with appropriate radiolabeled probes, a 4.1 kb fragment was seen in the case of wild-type *M*. *smegmatis* mc^2^155 cells. The predicted 3.14 kb and 2.09 kb fragments were observed in the *ΔrecX* and *ΔrecAΔrecX* mutants respectively (Fig. 3B,C). Both these bands are smaller than 4.1 kb, indicating that the *recX* and *recArecX* genes have been deleted by allelic replacement. The frequency of allelic exchange with respect to *recX* and *recArecX* was in the range of 70% and 80% respectively.

**Figure 4:**
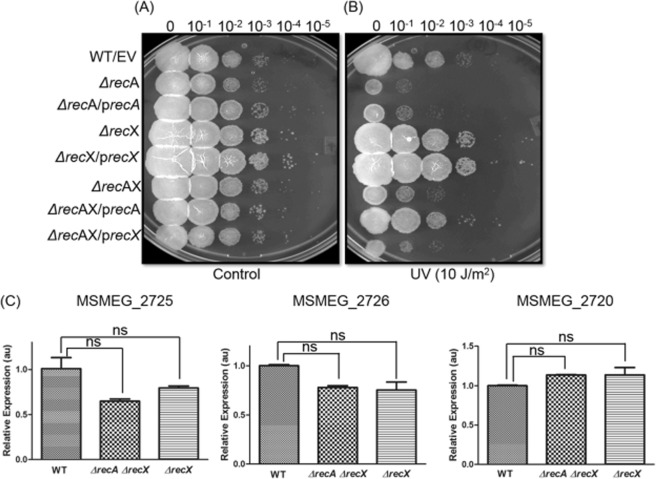
Insertion of a hygromycin resistance gene into *M*. *smegmatis* mc^2^155 *recA-recX* locus exerts no polar effect. The p*recA* and p*recX* denote pVV16-vector bearing one functional copy of either *recA* or *recX* gene under the control of the Hsp60 constitutive promoter. EV denotes pVV16 empty vector. *precA* and *precX* indicate plasmids bearing wild-type copies of *M*. *smegmatis recA* and *recX* genes respectively. These have been transformed into the indicated wild-type and mutant strains. Complementation of *M*. *smegmatis* mc^2^155*ΔrecA ΔrecX* and *ΔrecX* mutant strains for aerobic growth (panel A) and sensitivity against UV irradiation (panel B) with plasmids bearing wild-type alleles of *M*. *smegmatis recA* or *recX*. (**C**), quantitative real-time PCR analysis of genes around the *recA-recX* locus of *M*. *smegmatis* mc^2^155 wild-type and mutant strains. The error bars represent the standard error of the mean calculated from 3 independent replicates. Significant differences are indicated by ns, not significant.

One caveat of this analysis is that the observed effects of *ΔrecX* and *ΔrecAΔrecX* mutations could result from the polar effects of hygromycin-resistance gene insertion. In general, genetic alterations around the *recA-recX* locus could lead to polar effects on the expression of genes downstream of *recA-recX* locus. Two independent experiments were carried out to investigate the potential polar effects. First, the *ΔrecX* and *ΔrecAΔrecX* mutants were complemented with the functional copies of the *recA* and *recX* genes. The transformants were evaluated for their ability to grow in a standard culture medium and protect mutant cells against UV irradiation. Ten-fold serial dilutions were spotted on 7H10 agar plates and analyzed. As shown in Fig. 4A,B, the wild-type *recA* partially complemented the *ΔrecA* and *ΔrecAΔrecX* mutant strains, as deduced from their ability to support growth and provide protection against UV irradiation. However, wild-type *recX* failed to rescue the phenotype of *ΔrecAΔrecX* mutant strains observed upon DNA damage induction with UV irradiation. Since, deletion of *recX* had no discernible effect on its growth under normal and DNA damaging conditions, *ΔrecX* and its corresponding complemented strain showed similar growth as that of wild-type.

Second, we assessed the expression of two *M*. *smegmatis* mc^2^155 genes, *gluD* (MSMEG_2725) and *glnH* (MSMEG_2726), located downstream of the *recA recX* locus in the *ΔrecA ΔrecX* and *ΔrecX* mutant strains in comparison with the wild-type strain. The *M*. *smegmatis hyd2* (MSMEG_2720) gene located upstream of the *recA-recX* locus was used as a positive control. The total RNA was prepared from the wild-type, *ΔrecA ΔrecX* and *ΔrecX* mutant strains. The relative abundances of the gene transcripts were determined by quantitative RT-qPCR and normalized to that of the constitutively expressed chromosomal 16S rRNA gene. The expression levels were measured from cells grown in the late exponential growth phase. In all cases, the gene expression profiles of both the upstream and downstream ORFs were similar for the wild-type and mutant strains (Fig. 4C). Taken together, these results exclude the possibility that insertion of the hygromycin-resistance gene causes polar effects.

### The *M*. *smegmatis ΔrecA* and *ΔrecA ΔrecX* mutant strains show impaired growth and reduced cell yield relative to the wild-type strain

**Figure 5:**
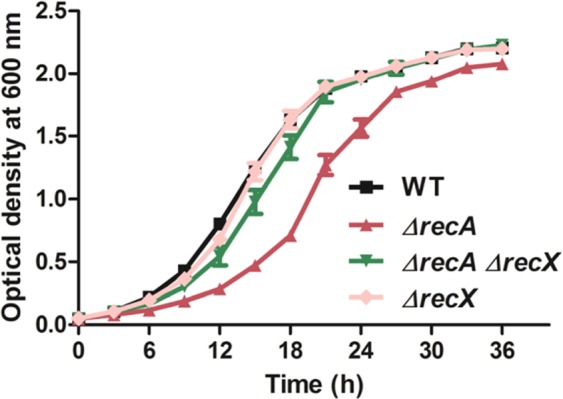
The kinetics of growth of *M*. *smegmatis* mc^2^155 wild-type, *ΔrecA* and *ΔrecA ΔrecX* and *ΔrecX* strains under normal growth conditions. Each data point is the mean of three independent experiments and the error bars represent standard deviations of the mean values.

To characterize the *M*. *smegmatis ΔrecX* and *ΔrecA ΔrecX* mutant strains, the growth profiles of the wild-type and knockout strains were measured in 7H9 Middlebrook broth at 37 °C (Fig. 5). The *recX* deletion had no discernible effect (compared to the wild-type strain) on the growth of *M*. *smegmatis*. Under similar conditions, *recA* deletion markedly increased the length of the lag phase and concomitantly reduced the exponential growth phase, suggesting that *recA* plays a role in the growth and viability of *M*. *smegmatis*. Furthermore, these results are consistent with the known function of RecA in the rescue of stalled or collapsed replication forks^71–73^. Because the *recA* and *recX* genes form a single operon, the impact of their deletion on *M*. *smegmatis* growth was investigated. The *ΔrecA ΔrecX* knockout strain exhibited a growth phenotype that was intermediate between the *ΔrecA* mutant and the wild-type strain; however the growth at late log and stationary phases was similar to that of *ΔrecA* and wild-type strains.

### Deletion of *recA*, but not *recX*, renders *M*. *smegmatis* more susceptible to DNA damaging agents

**Figure 6:**
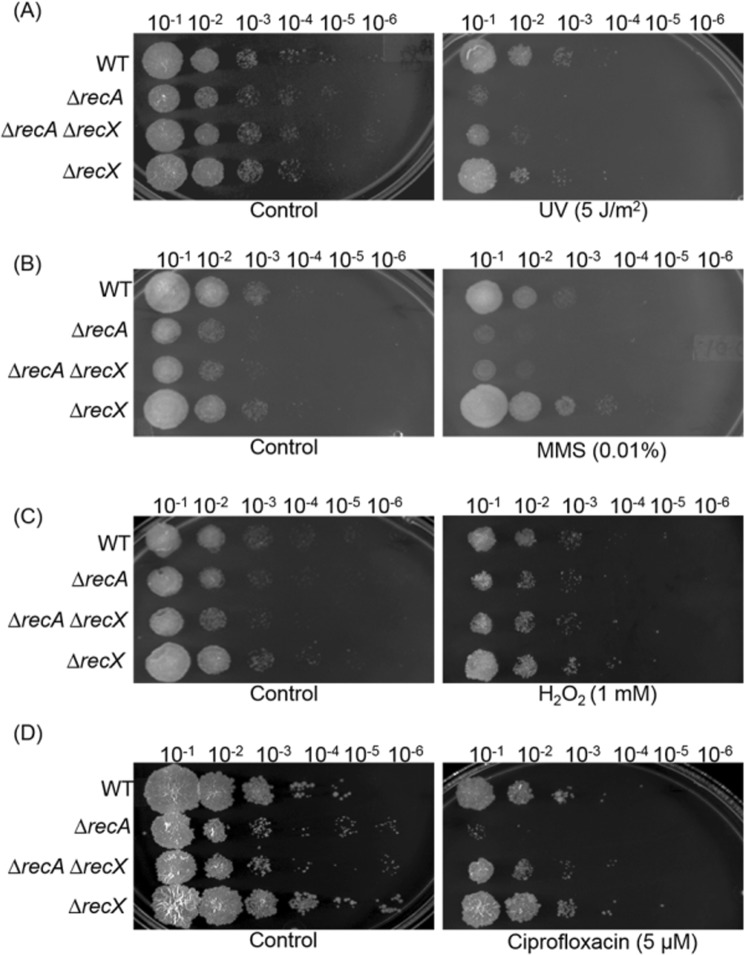
Effect of DNA-damaging agents on the survival of *M*. *smegmatis* mc^2^155 wild-type and *ΔrecA*, *ΔrecA ΔrecX* and *ΔrecX* **s**trains. The plates were exposed to: (**A**) five J/m^2^ UV light; (**B**) 0.01% MMS; (**C**) 1 mM H_2_O_2_ and (**D**) 5 μM ciprofloxacin. Control represents the growth of strains without any DNA damaging agent. The results are representative data (n = 3) from three independent experiments.

Similar to other eubacteria, the mycobacterial RecA protein plays a crucial role in regulating the SOS response upon DNA damage^74–77^. To test whether the absence of *recArecX* and *recX* affect the ability of *M*. *smegmatis* to effectively repair damaged DNA, the mutants were exposed to a range of agents with varied mechanisms of DNA damage. In these experiments, stress was induced by exposing the *ΔrecA*, *ΔrecX* and *ΔrecA ΔrecX* mutant strains to UV-irradiation, MMS, H_2_O_2_ or ciprofloxacin during the exponential phase (OD_600_ of 0.6) of the bacterial growth. After 3 h incubation, the cells were washed and their viability was assayed by spotting ten-fold serial dilutions of the cultures on Middlebrook 7H10 agar plates containing hygromycin (100 µg/ml). The most appropriate concentration/dose of the DNA-damaging agents was determined after evaluating the differences in cell viability between the strains using plate assays. In the absence of DNA-damaging agents, all the strains exhibited similar levels of cell viability (Fig. 6). In contrast, in the presence of DNA-damaging agents, *ΔrecA* strain showed a pronounced growth defect accompanied with 100-fold decrease in plating efficiency relative to the wild-type strain. This phenotype is consistent with the available literature. In contrast, the lethal effect of UV, MMS or ciprofloxacin, but not H_2_O_2_, was partially blocked by deletion of the *recX* gene in the *ΔrecA* strain. Although the basis for lack of H_2_O_2_ effect is not clear, the concentration used was probably not high enough to affect growth. Interestingly, the *ΔrecX* strain exhibited a slightly more resistant phenotype against all four DNA-damaging agents than the *ΔrecA ΔrecX* strain, indicating a possible gain-of-function due to loss of *recX* gene. Given these results, it is apparent that *recA* plays an active role in the SOS response and that the sensitivity of DNA damaging agents is slightly suppressed in *ΔrecX* strain.

### Survival after treatment with different concentrations of DNA-damaging agents

**Figure 7:**
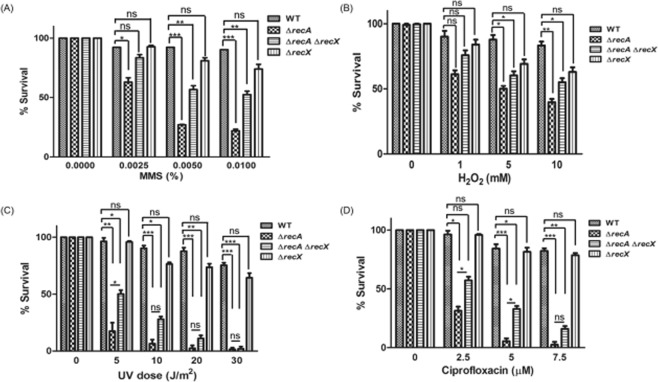
Survival of exponential-phase cultures exposed to a broad range of increasing concentrations of DNA damaging agents. The survival of *M*. *smegmatis* wild-type, *ΔrecA*, *ΔrecA ΔrecX* and *ΔrecX* strains was investigated by determining the number of CFUs. (**A**) The survival of cells treated with (**A**), MMS; (**B**) H_2_O_2_; (**C**) UV radiation and (**D**) ciprofloxacin. The error bars represent the standard error of the mean calculated from 3 independent replicates. Significant differences are indicated by *p < 0.05; **p < 0.01 and ***p < 0.001. ns, not significant.

Mycobacteria experience a number of adverse stress conditions, such as oxidative, nutritional and drug-induced stresses^78–82^. To further explore cell viability differences, experiments were carried out in which the cell viability was measured by colony forming units (CFUs). First, the viability of *M*. *smegmatis* mc^2^155 *ΔrecA*, *ΔrecX* and *ΔrecAΔrecX* mutants were tested in comparison with that of the wild-type by challenging them with increasing concentrations of MMS. The strains were grown in the presence of indicated concentrations of MMS, until the cultures reached an OD_600_ of 0.6. The cell viability was assayed by plating predetermined number of cells on 7H10 agar plates. The cells were judged alive if they were able to divide and form colonies. The mutants, unlike the wild-type strain, showed increased sensitivity to MMS in a concentration-dependent manner. The quantitative analysis indicated that the *ΔrecA* mutant exhibited relatively higher sensitivity to MMS, which increased with increasing concentrations of MMS (Fig. 7A). In the case of *ΔrecX*, the cells were ~2-fold less sensitive to MMS compared to the *ΔrecA* cells. On the other hand, the *ΔrecA ΔrecX* mutant showed more sensitivity to MMS in contrast to the *ΔrecX* mutant. The sensitivity of the *ΔrecX* mutant to MMS indicate that *recX* may have yet unidentified targets in addition to RecA. This assumption needs further investigation.

Next, the sensitivities of the *M*. *smegmatis* mc^2^155 *ΔrecA*, *ΔrecX* and *ΔrecA ΔrecX* mutant strains relative to the wild-type strain were determined by exposing them to increasing concentrations of H_2_O_2_. The results show that the *ΔrecA* mutant was less sensitive to this treatment (Fig. 7B). The deletion of *recX* alone, or deletion of the *recA-recX* locus, had only a minor effect on the viability and proliferation of mutant cells in response to H_2_O_2_ treatment. Notably, the *ΔrecA* and *ΔrecAΔrecX* mutant strains exhibited a sensitive growth phenotype to UV irradiation and ciprofloxacin that is significantly much more severe than to MMS or H_2_O_2_ (Fig. 7C,D). Furthermore, *ΔrecA* strain displayed greater sensitivity to UV irradiation and ciprofloxacin than the *ΔrecA ΔrecX* double mutant strain.

### The expression of *recA* and *recX* genes are induced by DNA damage

The regulatory elements upstream of the *recA* gene are not conserved in all mycobacterial species. For example, the *M*. *tuberculosis recA* gene is transcribed from two promoters: both are DNA damage-inducible, albeit through different mechanisms. The promoter P1 of *M*. *tuberculosis recA* (proximal to the start codon) can be induced following DNA damage independent of the LexA and RecA proteins. In contrast, the promoter P2 (located away from the *recA* start codon) is regulated by LexA, which is functionally analogous to the *E*. *coli recA* promoter^83–85^. Interestingly, the mechanism of DNA damage induction in *M*. *tuberculosis* is not fully conserved in *M*. *smegmatis*^46,75^. These findings emphasize the need to understand the expression and regulation of the *M*. *smegmatis recA* and *recX* genes and their roles in response to DNA-damaging agents.

**Figure 8:**
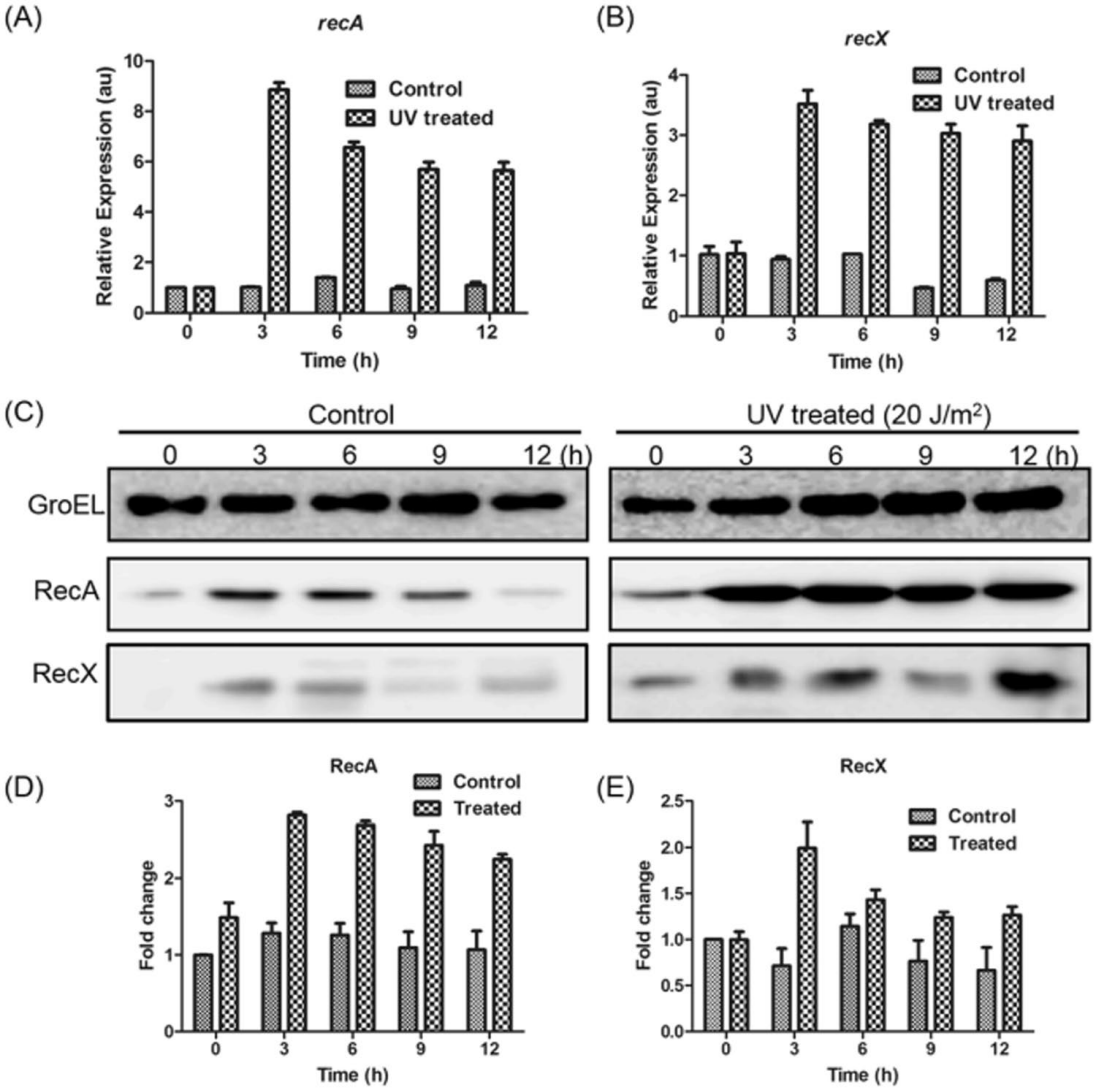
UV radiation induces the expression of *recA* and *recX* in *M*. *smegmatis*. (**A**) kinetics of accumulation of *recA* mRNA in control and following exposure to UV radiation; (**B**) kinetics of accumulation of *recX* mRNA in control and following exposure to UV radiation. (**C**) Representative Western blots showing the kinetics of accumulation of RecA and RecX proteins in control and UV-induced *M*. *smegmatis* cells. (**D**,**E**) Quantifications of Western blot data shown in panel C. The error bars represent the standard error of the mean calculated from 3 independent replicates.

As described above, hypoxia caused an increase in the expression of *M*. *smegmatis recA* and *recX* genes (Fig. 1). To further corroborate the notion that the expression of *M*. *smegmatis recA* and *recX* genes is damage-inducible, the kinetics of induction with and without DNA damage was determined using RT-qPCR. Total RNA samples were prepared from *M*. *smegmatis* cells harvested at 0, 3, 6, 9 and 12 h after exposure to UV light as well as from untreated cultures and analysed as described in the Methods section. The results revealed no significant differences between the *recA* and *recX* transcript levels in untreated cells. In contrast, a marked increase in the *recA* and *recX* mRNA transcripts was observed 3 h after exposure to UV radiation, and then decreased slightly thereafter. A comparison of the RT-qPCR data indicates important differences between the *recA* and *recX* transcript levels. Exposure to UV radiation led to a ~8-fold increase in *recA* mRNA over control, while *recX* by ~3-fold (Fig. 8A,B). Thus, although *recA* and *recX* mRNA exist in the same transcriptional unit, these results support the idea that the production and/or stability of *recX* mRNA transcript is subject to an additional posttranscriptional regulatory mechanism.

These results led us to perform additional experiments to determine the kinetics of accumulation of RecA and RecX proteins in *M*. *smegmatis* cells with or without DNA damage. Western blotting assays were performed on whole cell lysates using polyclonal antibodies raised against RecA and RecX (Fig. 8C). The quantification of Western blots showed that the cells contained significant quantities of both RecA and RecX proteins in uninduced cells (Fig. 8D,E). By comparison, a 2-fold increase in the abundance of both RecA and RecX (over control) was seen in cells 3 h after exposure to UV radiation and decreased thereafter. Importantly, the induction of RecA and RecX proteins exhibited a pattern reminiscent of that seen in cells under hypoxic conditions (Fig. 2), although the mechanisms by which they damage DNA are likely to be different.

### Concluding remarks

Numerous studies have demonstrated that *recA* and *recX* perform a broad range of functions related to DNA repair and recombination^7,10^. To our knowledge, the stimuli that activate the expression of *M*. *smegmatis recA* and *recX* genes have not been identified. In this study, the expression levels of these genes were assessed in cells at aerobic growth and in response to various stress conditions. In *M*. *smegmatis*, similar to many bacterial species, *recA* and *recX* belong to the same operon, and the *recX* gene is located immediately downstream of the *recA* gene, and share overlapping coding regions^86^. It was found that DNA-damaging agents induced the expression of both genes to different extents; however, the expression ratios follow a similar pattern. Interestingly, the levels of both RecA and RecX remain high under stress conditions compared to aerobic growth conditions.

Several studies have demonstrated alternative roles for *recX* in recombinational DNA repair promoted by RecA. While RecX protein physically interacts with RecA, and functions as a potent inhibitor of all known functions of the latter in many bacterial species^14,46,48^, it potentiates homologous recombination in *N*. *gonorrhoeae* and *B*. *subtilis*^25,26^. To gain insights into the role of *recX* in stress response in mycobacteria, knockout mutants of *M*. *smegmatis recX* and *recArecX* were constructed. Interestingly, the deletion of *recX* in *M*. *smegmatis* resulted in a slightly more resistant phenotype against all four DNA-damaging agents, indicating a possible gain-of-function due to loss of *recX* gene. The molecular basis of this effect is not clear, which seems worthwhile to explore in future studies. Whilst our analysis was mainly focused on the genetic interaction between *recA* and *recX* in recombinational DNA repair pathway, interestingly, *recX* seems to play an important role in bacterial growth. In summary, these findings are consistent with the idea that *M*. *smegmatis recX* plays an important role in DNA repair/recombination processes under adverse conditions, perhaps by regulating the detrimental effects of a subset of the yet unknown genes.

#### Abbreviations used are

DTT, dithiothreitol; EDTA, ethylene diamine tetraacetic acid; kb, kilobase; MMS, methylmethane sulfonate; ODN, oligonucleotide; PAGE, polyacrylamide gel electrophoresis; PVDF, polyvinylidene difluoride membrane; RT-qPCR, reverse transcription quantitative polymerase chain reaction; SDS, sodium dodecyl sulphate; ssDNA, single-stranded DNA; SSC, 0.15 M NaCl-0.015 M sodium citrate (pH 7.0) buffer.
